# Quantification of Metal(loid)s in Lubricating Eye Drops Used in the Treatment of Dry Eye Disease

**DOI:** 10.3390/molecules28186508

**Published:** 2023-09-07

**Authors:** Marcelo de Oliveira, Elaine S. de Pádua Melo, Thaís Carvalho da Silva, Carla Maiara Lopes Cardozo, Igor Valadares Siqueira, Mariana Pereira Hamaji, Vanessa Torres Braga, Luiz Fernando Taranta Martin, Alessandro Fonseca, Valter Aragão do Nascimento

**Affiliations:** 1 Graduate Program in Health and Development in the Central-West Region of Brazil, Federal University of Mato Grosso do Sul, Campo Grande 79079-900, Brazil; mdeoliveira@gmail.com (M.d.O.); nutricarlalopes@gmail.com (C.M.L.C.); nessatorresb@hotmail.com (V.T.B.); 2 Group of Spectroscopy and Bioinformatics Applied to Biodiversity and Health, Postgraduation Program in Health and Development in the Midwest Region, School of Medicine, Faculty of Medicine, Federal University of Mato Grosso do Sul, Campo Grande 79079-900, Brazil; elainespmelo@hotmail.com (E.S.d.P.M.); tata.silva1772@gmail.com (T.C.d.S.); igorvaladares@gmail.com (I.V.S.); marihamaji@gmail.com (M.P.H.); luiztaranta@gmail.com (L.F.T.M.); acfms2005@gmail.com (A.F.)

**Keywords:** heavy metals, metalloids, lubricating eye drops, dry eye disease

## Abstract

The aim of the study was to evaluate the presence of metal(loid)s in lubricating eye drops used in the treatment of dry eye disease. The concentrations of Al, As, Ba, Cd, Co, Cu, Cr, Pb, Fe, Mg, Mn, Mo, Ni, Se, V, and Zn were determined in 19 eye drop samples using inductively coupled plasma optical emission spectrometry (ICP OES). The limit of detection (LOD) and limit of quantification (LOQ) values for the quantified elements ranged from 0.0002–0.0363 (mg/L) and 0.0007–0.1211 (mg/L), respectively. High values of concentrations of Al (2.382 µg/g), As (0.204 µg/g), Ba (0.056 µg/g), Cd (0.051 µg/g), Co (1.085 µg/g), Cr (0.020 µg/g), Cu (0.023 µg/g), Fe (0.453 µg/g), Mg (24.284 µg/g), Mn (0.014 µg/g), Mo (0.046 µg/g), Ni (0.071 µg/g), Pb (0.049 µg/g), Se (0.365 µg/g), V (0.083 µg/g), and Zn (0.552 µg/g) were quantified in samples of eye drops with and without preservatives. The concentrations of As (5 samples) and Cd (3 samples) were higher than those allowed by the Brazilian Pharmacopoeia for impurities (parenteral use). The value of Co content (µg/g) in a sample was higher than the value established by the International Council for Harmonization of Technical Requirements for Pharmaceuticals for Human Use (ICH Q3D (R2)) in the parenteral route. The daily eye drop instillation exposure (µg/day) was below the values from the parenteral-permitted daily exposure (PDE) set by the ICH Q3D guideline (R2). The presence of heavy metals in eye drops is an alert to regulatory agencies in several countries so that control and inspections can be carried out.

## 1. Introduction

According to the World Health Organization (WHO, 2019), eye conditions that do not typically cause vision impairment, such as dry eye (DE), must not be overlooked [1]. Therefore, several countries have sought solutions to prevent and treat this disease [2]. Dry eye disease, also called dry eye syndrome, dysfunctional tear syndrome, and keratitis sicca, is a multifactorial disease of the ocular surface characterized by the loss of tear film homeostasis [3,4], which affects millions of people [4], with adults over 40 years of age and women being the most affected [5]. The most common causes of dry eye disease are age, menopause, hormonal changes, autoimmune diseases, inflamed eyelid glands, and allergic eye diseases. Further causes include the constant or excessive use of computers, exposure to air conditioning, the use of contact lenses, and the ingestion of drugs, such as benzodiazepines, antidepressants, antihistamines, and analgesics [3].

The diagnosis of DE is made from the identification of the symptoms and clinical tests. However, this disease has multiple symptoms and signs that often do not correlate with one another. As a result, greater attention is being paid to diagnosing and imaging technologies to assess the type and severity of dry eye disease [5,6,7]. Conventional treatments incorporate lubricating eye drops or artificial tears, which can be effective in mild and moderate cases [8]. In addition, lubricating eye drops have different levels of osmolarity, pH, and viscosity; however, they contain the same aqueous component that is important in lubricating the ocular surface [9].

The eye drops used in dry eye disease may contain preservatives in order to avoid contamination of bacteria in the vials after opening [10]. Although, chronic use of preservatives can cause significant damage to the eye tissues [11]. On the other hand, lubricating eye drops without preservatives can minimize the effects of irritation and ocular toxicity; however, they are expensive and the bottle is difficult to handle, especially for elderly patients [12].

Previous studies have reported that metals and metalloids play an important role in the pathogenesis of several ophthalmological disorders, such as Cd, Cu, Fe, Hg, Ni, and Zn in glaucoma [13,14], as well as the elements As, Cd, Co, Cr, Cu, Fe, Hg, Ni, and Pb in age-related macular degeneration (AMD) [15,16,17], Al, Bi, Cd, Cs, Fe, Pb, Te, and Tl in cataracts [18,19], and Cd, Hg, and Pb in dry eye disease [20,21,22,23,24]. Furthermore, there is a significant level of heavy metals in the aqueous humor [14,25], as well as in the lens [19,25,26], and in the pigment epithelium of the retina and choroid [17,27]. However, clinical studies on the toxicity of heavy metals and metalloids in eye drops with preservatives and without preservatives are scarce.

The impurities present in eye drops can be organic, inorganic, and residual solvents. Most of these impurities occur due to manufacturing processes, degradation, storage conditions, excipients, or contamination [28,29,30]. In this context, some countries have pharmacopoeias that establish quality parameters and analysis methods for supplies and medicines [31,32,33]. However, in the absence of information in their pharmacopoeias, some countries resort to the guidelines for elemental impurities (ICH Q3D), which are established by the International Council for Harmonization of Technical Requirements for Pharmaceuticals for Human Use (ICH) [34,35,36].

Since metals have various oxidation states, their toxicities are different [37]. Cr (III) is essential to health human, while Cr (VI) is highly toxic [38]. In addition, pentavalent methylated arsenicals are less toxic, while trivalent methylated species are highly cytotoxic [38]. Various transition metal ions are toxic and produce various types of diseases in the body. Metal ions are present in water, soil, sediment, and blood samples and their valence state plays a crucial role for absorption and dynamics behavior in the biological context [37,39]. In light of the above, it has been observed that more research is needed to investigate the values of the concentrations of metal(loid)s in various types of medication and compare them with the values established by the regulatory agencies.

The ICH Q3D guideline, currently in version 2 (R2), presents a process to assess and control elemental impurities in the drug product administered orally, parenterally, and inhaled; that is, it establishes the limits of permitted daily exposure (PDE) in µg/day for each element of toxicological concern and the concentration-permitted levels of impurities in drugs, drug substances, and excipients [40,41,42]. However, to date, permitted concentrations and daily exposure of metal(loid)s for the ophthalmic route have not been established [31,40,43].

Motivated by the results published in Refs. [13,14,15,16,17,18,19,20,21,22,23,24], who demonstrated that potential risks to ocular health may be related to the presence of heavy metals, our study aimed to evaluate, for the first time, the presence of Al (aluminum), As (arsenium), Ba (barium), Cd (cadmium), Co (cobalt), Cr (chromium), Cu (copper), Fe (iron), Mg (magnesium), Mn (manganese), Mo (molybdenum), Ni (nickel), Pb (lead), Se (selenium), V (vanadium), and Zn (zinc) in Brazilian lubricating eye drops with and without preservatives used in the treatment of dry eye disease. In addition, the contents of chemical elements in the studied eye drop samples were compared with the values of allowed concentrations of permitted concentration of elemental impurities established by the Brazilian Pharmacopoeia (BP) [31], as well as elemental impurity values set by the ICH Q3D (R2) [40], and PDE values recommended by the ICH Q3D guideline (R2) in the parenteral route [40].

## 2. Results

The results were presented as follows: Section 2.1 outlines the results of the analyte addition and recovery test; the result of the calibration curve is shown in Section 2.2; in Section 2.3, data on the concentrations of heavy metals and metalloids quantified (µg/g) in samples of lubricating eye drops and the comparison with the values of permissible concentrations of elemental impurities established in the parenteral route of Brazilian Pharmacopeia (BP) [31] and ICH guideline Q3D (R2) [40] are presented. The latter data (Section 2.4) provides data on the exposure to heavy metals and metalloids during the daily instillation of lubricating eye drops in the eyes (µg/day) and the comparison with the PDE values established by the ICH Q3D guideline (R2) via the parenteral route.

### 2.1. Analyte Addition and Recovery Test

A spike was generated by adding a known amount (0.5 mg/L) of analyte to a sample, testing the spiked sample. The recovery results were as follows: Al 91.37%, As 90.86%, Ba 95.38%, Cd 96.12%, Co 90.57%, Cr 99.05%, Cu 91.69%, Fe 96.19%, Mg 97.13%, Mn 95.31%, Mo 90.23%, Ni 92.70%, Pb 95.85%, Se 97.10%, V 91,12%, and Zn 98.17%.

### 2.2. Calibration Curves

The range values of the detection limits (LOD), quantification limits (LOQ), and correlation coefficients (R^2^) for the detected elements were 0.0002–0.0363 (mg/L), 0.0007–0.1211 (mg/L), and 0.9968–0.9996, respectively. Table 1 presents the parameters of the calibration curve and the values obtained for the LOD, LOQ, and R^2^.

### 2.3. Concentration of Metal(loid)s in Lubricating Eye Drops

**Figure 1:**
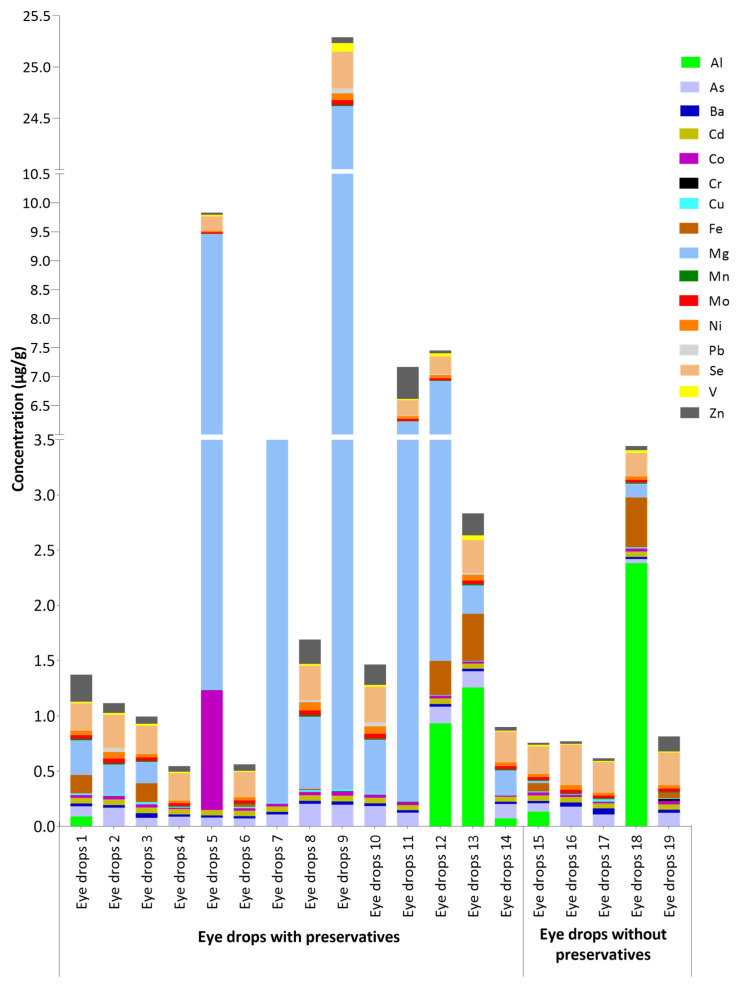
Values of concentrations of metal(loid)s in lubricating eye drops with and without preservatives.

In this study, the concentrations of metal(loid)s in 19 commercial samples of lubricating eye drops used in the treatment of dry eye disease in Brazil were analyzed using optical emission spectrometry with inductively coupled plasma (ICP OES). A total of fourteen samples with preservatives (samples 1–14) and five without preservatives (samples 15–19) were analyzed. Table 2 shows the concentrations of Al, As, Ba, Cd, Co, Cr, Cu, Fe, Mg, Mn, Mo, Ni, Pb, Se, V, and Zn quantified in lubricating eye drops with preservatives and without preservatives compared with the permitted concentrations of elemental impurities established by the BP [31] and ICH Q3D (R2) guideline for parenteral administration [40]. Figure 1 shows the values of metal(loid) concentrations quantified in the eye drop samples with and without preservatives. The concentration of Al in the eye drop samples 2–11, 16, 17, and 19, Cr in samples 1–8, 10, and 12–18, Fe in samples 2, 4, 5, 7, 9–11, 14, 16, and 17, Mg in samples 4, 6, 17, 18, and 19, and Pb in samples 1, 3–7, and 14–19 are all below the limit of detection (<LOD).

### 2.4. Exposure to Metal(loid)s by Daily Instillation of Lubricating Eye Drops

**Figure 2:**
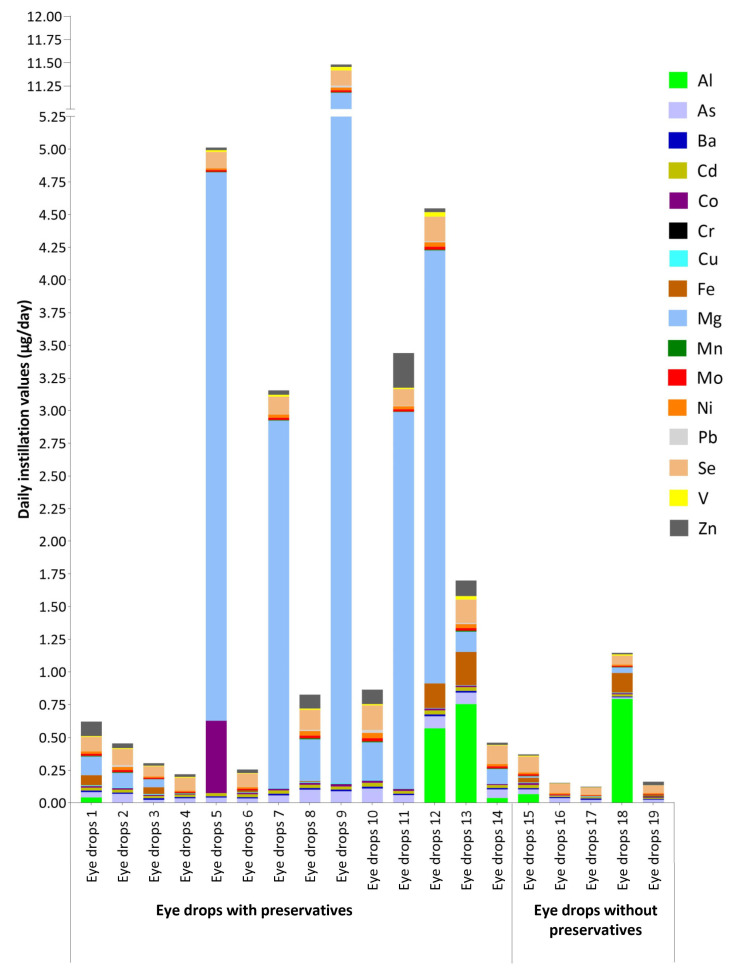
Values of daily instillation of lubricating eye drops with and without preservatives.

Exposure to metal(loid)s through the daily instillation of lubricating eye drops was obtained by multiplying the concentration of quantified elements in the eye drops (Table 2) by the maximum daily dose of the sample [44]. In addition, the results obtained regarding the exposure to metal(loid)s through the daily instillation of lubricating eye drops were compared with the PDE for elemental impurities of the ICH Q3D (R2) guideline for the parenteral route (Table 3). Figure 2 shows the values obtained through the daily instillation of lubricating eye drops in the eyes (µg/day) with and without preservatives.

## 3. Discussion

Spike tests generated information about the recovery of the spiked samples. Since there are no certified reference metal and metalloids samples in eye drops, spike recovery tests must be used for quality control. The values of LOD and LOQ are displayed in Table 1. As can be seen, the LODs and LOQs allowed for the determination of both metals and metalloids at the required levels based on the IUPAC [45].

The concentrations of Al, As, Ba, Cd, Co, Cr, Cu, Fe, Mg, Mn, Mo, Ni, Pb, Se, V, and Zn in lubricating eye drops from different manufacturers (Table 2) decreased in values as follows:

eye drops 1 (with preservatives): Mg > Zn > Se > Fe > As > Al > Cd > Ni > Mo > Ba > Co > Cu > V > Mn;
eye drops 2 (with preservatives): Se > Mg > As > Zn > Ni > Cd > Mo > Pb = Co > Ba > V > Mn > Cu;
eye drops 3 (with preservatives): Se > Mg > Fe > As > Z > Cd > Ba > Mo > Ni > Co > Cu > V > Mn; eye drops 4 (with preservatives): Se > As > Zn > Cd > Mo > Ni > Ba > Co = V > Cu > Mn;
eye drops 5 (with preservatives): Mg > Co > Se > As > Cd > Zn > V > Mo > Ni > Ba > Mn > Cu;
eye drops 6 (with preservatives): Se > As > Zn > Cd > Mo > Ni > Co > Fe > Ba > V = Cu > Mn;
eye drops 7 (with preservatives): Mg > Se > As > Zn > Ni = Cd >Mo > V > Co > Ba > Mn > Cu;
eye drops 8 (with preservatives): Mg > Se > Zn > As > Ni > Cd > Mo > Co > Ba > Pb > V > Cu > Mn > Fe;
eye drops 9 (with preservatives): Mg > Se > As > V > Ni > Zn > Cd > Pb > Mo > Co > Ba > Cu > Mn > Cr;
eye drops 10 (with preservatives): Mg > Se > Zn > As > Ni > Cd > Mo > Pb > Co > Ba > V > Mn > Cu;
eye drops 11 (with preservatives): Mg > Zn > Se > As > Ni > Cd > Mo > Co > Ba > V > Pb > Mn > Cr > Cu;
eye drops 12 (with preservatives): Mg > Al > Fe > Se > As > Ni > V > Zn = Cd > Mo > Co = Ba > Pb > Mn > Cu;
eye drops 13 (with preservatives): Al > Fe > Se > Mg > Zn > As > Ni > Cd > V > Mo > Ba > Co > Pb > Mn > Cu;
eye drops 14 (with preservatives): Se > Mg > As > Al > Cd > Ni = Zn > Mo > Ba > Co > Mn > V > Cu;
eye drops 15 (without preservatives): Se > Al > Fe > As > Cd > Co > Ni = Mo > Ba = Mg > Zn > V > Cu = Mn;
eye drops 16 (without preservatives): Se > As > Cd >Ni > Ba > Mo > Zn > Co > V > Mn > Cu;
eye drops 17 (without preservatives): Se > As > Ba > Cd > Mo = Ni > Zn > Cu > Co > V > Mn;
eye drops 18 (without preservatives): Al > Fe > Se > Mg > Cd > Zn > As > Ni > Co > V > Mo > Ba > Cu = Mn;
and eye drops 19 (without preservatives): Se > Zn > As > Fe > Cd > Co > Ba = Ni > Mo > Cr > V > Mn > Cu.

Values of the concentrations of metal(loid)s quantified in lubricating eye drops with preservatives and without preservatives are shown in Figure 1. Higher concentrations were obtained for several elements, such as Mg, Al, and Co. The statistical results for all data in Table 2 are shown in Supplementary Materials Table S1. According to the ANOVA, there is a statistically significant difference in the concentration means of Al, As, Ba, Cd, Co, Cr, Cu, Fe, Mg, Mn, Mo, Ni, Pb, Se, V, and Zn in all eye drops with preservatives and without preservatives. Tukey’s test identifies which means are significantly different from the others. In Table S1 (Supplementary Materials), when the letters are the same, there are no statistical differences; on the other hand, when the letters are different, there is a statistical difference between the values.

Figure 2 shows the values of exposure to metal(loid)s through the daily instillation of lubricating eye drops with and without preservatives. Elevated exposure values were deemed to be due to higher concentrations of the elements Mg, Al, and Co.

Table S2 (Supplementary Materials) shows the statistical test used in view of the results of Table 3. According to the ANOVA statistical test and Tukey’s test, there are significant differences in the average values of daily exposure to metal(loid)s between the samples of lubricating eye drops with preservatives and the samples without preservatives (*p* < 0.05) (Table S2 in the Supplementary Materials). For all values of daily instillation with the same letter, the difference between the means is not statistically significant.

According to Table 2, the content of Al ranged from 0.073 µg/g to 2.382 µg/g. There were levels of Al detected in eye drop samples 1, 12, 13, and 14 with preservatives, and in eye drops 15 and 18 without preservatives. As shown in Table 2, there were no values of Al detected in the parenteral drugs established by the BP and ICH Q3D guideline (R2). On the other hand, exposure to Al through the daily instillation of lubricating eye drops in Table 3 ranged from 0.037 µg/day (eye drops 14 with preservatives) to 0.793 µg/day (eye drops 18 without preservatives). There were no PDE values for the elemental impurities established by the ICH Q3D (R2) parenteral guideline. Nevertheless, Al can potentiate oxidative, inflammatory events and cause eventual tissue damage [46]. According to research involving animals, intraperitoneal application of aluminum chloride causes retinal toxicity and loss of the outer segments of photoreceptors [47].

The concentration of As in the eye drop samples ranged from 0.036 µg/g to 0.204 µg/g (Table 2). In eye drops 12 (0.147 µg/g) and 13 (0.147 µg/g), both with preservatives, the concentration values for As were close to the permitted concentrations of elemental impurities established by the BP for the parenteral route (0.15 µg/g), while the contents in eye drops 2 (0.169 µg/g), 8 (0.204 µg/g), 9 (0.195 µg/g), and 10 (0.184 µg/g) with preservatives and eye drops 16 without preservatives (0.178 µg/g) were all above the concentration value defined in the BP for parenteral use (See Table 2). In addition, all eye drops had concentrations of As below the limit established by the ICH Q3D guideline (R2) for this element in the parenteral route (1.5 µg/g). According to Table 3, the values of exposure to As due to the daily instillation of eye drops ranged from 0.012 µg/day (eye drops 18 without preservatives) to 0.109 µg/day (eye drops 10 without preservatives). The values of exposure to As in all eye drop samples were below the PDE limits established by the ICH Q3D guideline (R2) (15 µg/day). However, although the value of daily exposure to As through the instillation of lubricating eye drops is below the value established by the ICH Q3D guideline (R2), chronic exposure to this element can cause dermal effects, cardiovascular diseases, diabetes, cancers [48], eye irritation [49], conjunctivitis [50], and visual loss [51].

In Table 2, the content of Ba in the eye drop samples ranged from 0.021 µg/g to 0.056 µg/g. There are no Ba values established by the BP for impurities in parenteral drug administration. However, all eye drop samples showed concentrations of Ba below the values allowed by the ICH Q3D guideline (R2) via the parenteral route (70 µg/g). According to Table 3, the exposure to Ba caused by the daily instillation of lubricating eye drops ranged from 0.006 µg/day (eye drops 19 without preservatives) to 0.015 µg/day (eye drop samples 10 and 12 with preservatives); moreover, these values are below the PDE (700 µg/day) established by the ICH Q3D guideline (R2) in the parenteral route. The exposure to barium compounds can cause hypokalemia, cardiovascular diseases, muscle weakness and paralysis [52,53], and eye pain [54], as well as a toxic ocular inflammatory reaction (toxic anterior segment syndrome—TASS) [55]. In rabbits, topical administration of Ba resulted in mild skin irritation and severe eye irritation [56].

The concentration of Cd in the eye drop samples ranged from 0.044 µg/g to 0.051 µg (Table 2). The Cd contents in eye drops 1 (0.049 µg/g), 6 (0.048 µg/g), 7 (0.048 µg/g), 8 (0.049 µg/g), 10 (0.049 µg/g), and 12 (0.049 µg/g) with preservatives, and eye drops 15 (0.049 µg/g) and 16 (0.048 µg/g) without preservatives were close to the concentration values of impurities in drugs allowed by the parenteral BP (0.05 µg/g), while the concentrations in eye drops 2, 3, and 9 (0.051 µg/g) with preservatives are above the allowed concentration of impurities established by the BP for parenteral use. All samples showed cadmium concentrations below the concentration value allowed by the ICH Q3D guideline (R2) parenterally (0.2 µg/g) (Table 2). Regarding Cd exposure, daily instillation values ranged from 0.009 µg/day (eye drops 16,17, and 19 without preservatives 16) to 0.030 µg/day (eye drops 12 with preservatives); all daily instillation values were below the PDE defined by the ICH Q3D guideline (R2) (2 µg/day) (Table 3). In the body, Cd can cause health risks, such as acute and chronic intoxication, pathological changes in the organs, and cardiovascular, kidney, bone, liver disease, cancer [57], and eye problems [13,16,17,18], such as cataracts [26] and glaucoma, especially in men [13]. Park et al. [15] suggested that Cd, Pb, and Hg may negatively influence AMD. On the other hand, the study by Liou et al. [20], involving 59 welders and 25 administrative staff from a shipyard in northern Taiwan, concluded that Cd found in urine and Pb in welders’ toenails (exposed group) may be related to dry eye disease. In fact, according to a study carried out by Choi et al. [23], considering the exposure of women to phthalates and heavy metals and diagnosed and non-diagnosed with dry eye disease, women diagnosed with dry eye disease have higher concentrations of Cd in their urine and blood compared to those not diagnosed.

As shown in Table 2, the content of Co in the eye drops ranged from 0.013 µg/g to 1.085 µg/g. In addition, there are no limits for Co concentrations established by the BF. Only eye drops 5, containing preservatives, had a concentration value greater than that established by the ICH Q3D guideline (R2) parenterally (0.5 µg/g). In Table 3, the values of exposure to Co considering the daily instillation of eye drops ranged from 0.003 µg/day (eye drops 17 without preservatives) to 0.554 µg/day (eye drops 5 with preservatives). The PDE values for elemental impurities established by the ICH Q3D guideline (R2) in the parenteral route are 5 µg/day. After comparison, it was observed that the daily instillation values considering Co in Table 3 were below those established by the ICH Q3D guideline (R2). However, excessive levels of Co in the body can cause cancer [58], peripheral neuropathy, hearing loss, cognitive deficits, cardiomyopathy, hypothyroidism, and visual changes [59,60]. Junemann et al. [16] quantified the levels of selected metal ions in the aqueous humor of eyes affected by AMD using flow injection inductively coupled plasma mass spectrometry (FI-ICP-MS) and observed that patients with AMD had significant levels of Cd, Co, Fe, and Zn, in addition to reduced levels of Cu, when compared to patients without AMD. One study reported that a 39-year-old woman presented with blurred vision in the left eye and bilateral ocular discomfort following the bilateral implantation of hip prostheses that had Co in their constitution [61]. In 2020, Garcia and collaborators published a case report of a 59-year-old woman who presented symptoms, such as blurred vision, white spots in both eyes, hypothyroidism, cardiomyopathy, and neuropathy, after reattachment of a hip prosthesis implant with a Co piece [59]. In both cited studies, the hypothesis was that implant-related Co toxicity occurred. Finally, Lim et al. [62] reported that elevated serum Co levels may be related to reversible and irreversible damage that leads to visual loss, such as neuropathy and atrophy of the optic nerve and abnormal electrophysiological functioning of the retinal tissue and the retinal pigment epithelium, in addition to abnormal choroidal perfusion.

As shown in Table 2, the content of Cr in eye drops 1–8, 10, 12–18, and 19 are below the detection limit. However, the Cr content in eye drops 9 (0.004 µg/g) and 11 (0.004 µg/g) with preservatives and eye drops 19 (0.020 µg/g) without preservatives were all below the permissible BP concentration (2.5 µg/g) and ICH guideline Q3D guideline (R2) through the parenteral route (110 µg/g). According to the results shown in Table 3, exposure to Cr ranged from 0.002 µg/day (eye drops 9 and 11 with preservatives) to 0.004 µg/day (eye drops 19 without preservatives). In Table 3, the exposure values for the instillation of eye drops considering Cr were all below the PDE for elemental impurities defined by the ICH Q3D guideline (R2) parenterally (1100 µg/day). Although these concentrations are low when compared to the limits established by the BP and ICH Q3D guideline (R2), exposure to Cr can cause hepatotoxicity, nephrotoxicity, dermatotoxicity, carcinogenicity [63], and inflammation of the digestive tract followed by necrosis, dermatitis, chronic painless ulcers, teeth and tongue am-amery, irritation of the mucous membranes, respiratory allergies, and cancer at the level of the respiratory system [64]. Exposure to hexavalent Cr can cause eye irritation, corrosion, and ulceration. [65,66].

The contents of Cu in the eye drops ranged from 0.002 µg/g (eye drops 14 with preservative) to 0.023 µg/g (eye drops 17 without preservative), being below the concentration allowed by the BP (25 µg/g) and ICH Q3D guideline (R2) parenterally (30 µg/g) (Table 2). Exposure to Cu due to the daily instillation of eye drops, whose values ranged from 0.001 µg/day (eye drops 11 and 14 with preservatives, and 16 and 19 without preservatives) to 0.007 µg/day (eye drops 1, 8 and 9 with preservatives), were below the PDE for elemental impurities defined by the ICH Q3D guideline (R2) in the parenteral route (300 µg/day) (Table 3). Cu plays a key role in the biochemistry of the human nervous system [67], being important for healing and slowing cataract growth [68]. On the other hand, intraocular foreign bodies containing Cu can cause inflammation, damage to the cell membranes and mitochondria in the retina [14], aseptic abscesses, cataracts, vitreous liquefaction and retraction, retinal damage and detachment, ocular hypotension [69], and corneal opacities [70].

In Table 2, the contents of Fe ranged from 0.013 µg/g (eye drops 8 with preservatives) to 0.453 µg/g (eye drops 18 without preservatives). However, there are no Fe concentration values in drugs allowed by the BP and ICH Q3D guideline (R2) parenterally. As shown in Table 3, exposure to Fe due to the daily instillation of eye drops ranged from 0.006 µg/day to 0.256 µg/day (drops 8 and 13 with preservatives). However, there are no Fe values established by the ICH Q3D (R2) parenteral guideline for parenteral Fe. This element is involved in several biological processes, but the cerebral accumulation or the decrease in intracellular Fe can impair the functioning of several functions in the central nervous system (CNS) and cause cell death [71]. Fe affects the retina [72] and its overload augments stage I and stage II of tumor promotion in murine skin [73]. Chronic accumulation of Fe in the retina can cause AMD and also influence other ocular conditions, such as hereditary aceruloplasminemia, pantothenate kinase-associated neurodegeneration, intraocular hemorrhage [74], and glaucoma [14].

The concentration of Mg ranged from 0.021 µg/g (eye drops 15 without preservatives) to 24.284 µg/g (eye drops 9 with preservatives). However, there are no Mg concentration values in drugs allowed by the BP and ICH Q3D guideline (R2) parenterally. According to Table 3, exposure to Mg due to the daily instillation of eye drops ranged from 0.010 µg/day (eye drops 15 without preservatives) to 11,025 µg/day (eye drops 9 with preservatives). There are no PDE values for Mg as elemental impurities established by the ICH Q3D (R2) guideline for the parenteral route. In addition, the exposure of eye drops 9 with preservatives was higher in relation to the other heavy metals and metalloids analyzed. In this study, the highest values found for concentration and exposure through the daily instillation of eye drops were in relation to Mg. In the human body, Mg is important for maintaining the structural and functional integrity of the lens, in addition to playing a significant role as a cofactor for enzymes involved in the production and hydrolysis of adenosine triphosphate (ATP). Mg deficiency leads to the accumulation of Ca in the lens, which can cause opacification, along with the release of nitric oxide that produces nitrogen free radicals that are capable of causing oxidative damage [25]. In toxic situations, the most common symptoms are diarrhea, nausea and vomiting, muscle weakness, and low blood pressure, but as levels increase there is a loss of deep tendon reflexes, blockages of the sinus or atrioventricular node, respiratory paralysis, and, eventually, cardiac arrest [75].

Mn levels in eye drops 5 (0.006 µg/g), 8, and 9 (0.014 µg/g) with preservatives are below the concentration value established by the BP for impurities in parenteral drugs (25 µg/g). However, there are no values for Mn established by the ICH Q3D (R2) guideline for parenteral administration. Table 3 shows that exposure to Mn due to the daily instillation of eye drops ranged from 0.001 µg/day in samples 16 and 17 without preservatives; on the other hand, in eye drops 8 and 13 with preservatives it was 0.007 µg/day. There are no PDE values for elemental impurities set out in the ICH Q3D (R2) parenteral guideline. However, in the human body, Mn is important for healing and in delaying cataract growth [68]; meanwhile under high concentrations, Mn can cause toxicity to the reproductive, cardiac, respiratory, and central nervous systems [64]. According to animal studies, there are toxic effects of manganese ion eye drops to the ocular anterior segment [76]. Khosla et al. [77] studied the effect of Mn on the retina of rabbits and found that it is potentially retinotoxic, causing a selective effect on photoreceptors and ganglion cells. In addition, studies involving humans proved that patients with age-related ocular disorders have higher Mn concentrations when compared to a control group [78].

The Mo content in eye drops ranged from 0.025 µg/g (eye drops 18 without preservatives) to 0.046 µg/g (eye drops 9 with preservatives); however, such values were below the permissible BP concentration (2.5 µg/g) and ICH Q3D guideline (R2) for the parenteral route (150 µg/g). Exposure to Mo considering the daily instillation of eye drops ranged from 0.005 µg/day (eye drops 17 without preservatives) to 0.026 µg/day (eye drops 10 with preservatives). Therefore, Mo exposure values (Table 3) were found to be below those of the PDE established by the ICH Q3D guideline (R2) for the parenteral route (1500 µg/day). However, in studies involving workers exposed to high levels of Mo, it was found that the inhalation of dust containing this metal can cause lung problems [79]. According to Khosla et al. [77], retinotoxic effects of molybdenum on rabbit retina were observed (in an experimental study). Results involving humans corroborate those published with animal models [77]. In fact, Ceylan et al. [78] found an elevated Mo level in patients with pseudoexfoliation syndrome. In another study, molybdenum trioxide, a soluble molybdenum compound, was found to have an irritating effect on the skin, as well as the mucous and eyes [80].

The concentration of Ni ranged from 0.023 µg/g to 0.071 µg/g, but these values were found to be below the concentration value of impurities in drugs established by the BP (2.5 µg/g) and ICH Q3D guideline (R2) for parenteral administration (2 µg/g) (Table 2). Considering the daily instillation of eye drops (Table 3), the results indicate that exposure to Ni ranged from 0.005 µg/day (eye drops 17 without preservatives) to 0.040 µg/day (eye drops 10 with preservatives). However, these daily eye drop instillation values were found to be below the PDE for Ni (20 µg/day) established by the ICH Q3D guideline (R2) for the parenteral route. Ni is essential for the human body and is part of enzymatic and hormonal activity, and aids in promoting the structural stability of biological macromolecules and metabolism. Its toxicity causes headaches, nausea, vomiting, apathy, diarrhea, fever, skin disorders, carcinomas in the nasal cavities and lower respiratory tract [64], and conjunctivitis [81]. According to an animal study, cellular anomalies in the retina of fish eyes exposed to Ni affect the primary functions of the retina and lead to visual loss or low vision [82]. A study with eyeballs from human cadavers found that the levels of Ni, As, Cd, Cr, and Pb were elevated in late AMD when compared to the control groups [17].

In Table 2, the concentration of Pb in the eye drop samples ranged from 0.009 µg/g (eye drops 11 with preservatives) to 0.049 µg/g (eye drops 9 with preservatives). However, this concentration was below the allowed concentration for impurities in drugs established by the BP (0.1 µg/g) and ICH Q3D guideline (R2) for the parenteral route (0.5 µg/g). According to these results, considering the daily instillation of eye drops (Table 3), the exposure to Pb that varied from 0.004 µg/day to 0.022 µg/day in eye drops 2, 8–12, and 13, all with preservatives, were below the PDE for Pb (5 µg/day) established by the ICH Q3D guideline (R2) in the parenteral route. However, the accumulation of Pb in the body can cause health risks and be associated with eye diseases [14,27,83], such as cataracts [18], AMD [15,17], and dry eye disease [20,24].

As shown in Table 2, the contents of Se ranged from 0.211 µg/g to 0.365 µg/g. There are no Se values for impurities in drugs established by the BP for the parenteral route. In addition, all eye drop samples had concentrations below the concentration allowed in the parenteral route (8 µg/g) established by the ICQ Q3D guideline (R2). Daily exposure to Se ranged from 0.055 µg/day (eye drops 17 without preservatives) to 0.188 µg/day (eye drops 12 with preservatives) (Table 3). Furthermore, the Se exposure values in Table 3 were all below the PDE for Se (80 µg/day) defined by the ICH guideline Q3D (R2) for the parenteral route. However, exposure to Se at high levels can cause selenosis, respiratory tract irritation, bronchitis, difficulty breathing, stomach pains, and coughing [84]. In studies with mice, it was observed that after topical application of selenium sulfide erythema, skin irritation, acanthosis, and severe skin damage occurred [85]. According to the results of the study by Bruhn et al. [86], there is a relationship between glaucoma and selenium levels in plasma and the aqueous humor. In another experimental study involving animals, it was verified that any amount of Se above the concentrations necessary for the synthesis of selenoproteins is toxic and can cause cataracts [87].

The concentration values of V in the eye drops ranged from 0.008 µg/g (eye drops 14 with preservatives) to 0.083 µg/g (eye drops 9 with preservatives) (Table 2). In addition, this concentration was below that allowed by the BP for impurities in drugs (2.5 µg/g) and by the ICH Q3D guideline (R2) for the parenteral route (1 µg/g). Table 3 shows that exposure to V due to daily instillation ranged from 0.002 µg/day (eye drops 16, 17, and 19 without preservatives) to 0.038 µg/day (eye drops 9 with preservatives). Furthermore, these values were found to be lower than those established by the ICH Q3D (R2) guideline for Se (10 µg/day). In situations of V poisoning, people may have symptoms, such as headaches, hand tremors, high blood pressure, green tongue, cough, palpitations, wheezing, ear and throat irritation [88], skin rash [89], eye irritation, and conjunctivitis [90]. Furthermore, studies have shown that inhalation of V produces damage to the retina detected through the damage markers GFAP (glial fibrillary acidic protein) and GS (glutamine synthetase) and might interfere with vision [91,92].

The concentration of Zn ranged from 0.019 µg/g (eye drops 15 without preservatives) to 0.552 µg/g (eye drops 11 with preservatives) (Table 2). However, there are no established concentration values for Zn in impurities in drugs established by the BP and ICH Q3D (R2) guideline for the parenteral route. Exposure to Zn ranged from 0.004 µg/day (eye drops 16 without preservatives) to 0.265 µg/day (eye drops 11 with preservatives) (Table 3). There are no PDE values for Zn established by the ICH Q3D guideline (R2) considering the parenteral route. Zn is essential for the immune system [64] and acts to prevent the progression of AMD in advanced stages [15,93], in addition to influencing muscle function and vision [83]. However, its excess in the lens may be related to the presence of high molecular weight proteins that are considered precursors of insoluble protein aggregates that cause lens opacification [25]. Ocular exposure to zinc salts can cause irritation, pain, corneal ulcerations, edema and burns, hyperemia, hemorrhage, bullous keratopathy, glaucoma, cataract formation, discrete gray spots on the lens, tearing, significant reduction in acuity visual with hemorrhage, and conjunctival inflammation [94].

As we observed in the results and discussion above, although the concentrations of several elements are below the limit values that have been established by regulatory agencies, such as the BP and ICH Q3D (R2), it does not mean that their use is safe. Metals and metalloids can be dangerous for human health as they have a long biological half-life and are not biodegradable, and some are toxic even at very low concentrations [95,96,97].

This study shows that there is a presence of toxic metals in eye drops used in the treatment of dry eye; therefore, regulatory agencies must establish a PDE level in micrograms per day (µg/day) through the instillation of lubricating eye drops in the eyes.

## 4. Materials and Methods

### 4.1. Sample Collection

In this study, a total of 95 eye drop samples from different manufacturers were purchased in December 2021 from pharmacies in the city of Campo Grande, state of Mato Grosso do Sul, Brazil. Only samples of lubricating eye drops with a volume of 10 mL and available in drops were selected, with 5 samples of manufacturers without preservatives (25 samples from each batch and same eye drop manufacturer) and 14 samples of manufacturers with preservatives (70 samples from each eye drop manufacturer). From each company, 5 copies of the same batch were purchased; however, samples from each batch and from the same company were mixed to obtain a representative sample for analysis.

### 4.2. Preparation for the Analysis

All materials used in the study, such as Falcon glassware and plastic tubes, underwent a chemical demineralization process before being used. The materials were placed in a solution of Extran (5%, *v*/*v*) and nitric acid (10% concentration, Merck, Darmstadt, Germany) for a minimum period of 24 h, following which they were rinsed in ultrapure water and dried in an oven at 42 °C.

### 4.3. Acid Digestion of Samples

Approximately 0.5 mL of each sample was placed in a glass test tube, followed with the additions of 2.0 mL of HNO_3_ (65%, Merck, Darmstadt, Germany), and 1.0 mL of H_2_O_2_ (35%, Merck, Darmstadt, Germany).

The samples were digested in an open digestion system for 40 min at a temperature of 80 °C. Subsequently, all samples were transferred from glass test tubes to Falcon tubes, where 2.5 mL of ultrapure water (conductivity 18.2 MΩcm (Millipore), Biocel, Germany) was added.

All steps of the digestion analysis were performed in triplicate and the analytical blanks were prepared following the same procedure used in the samples.

### 4.4. Elementary Analysis Using ICP OES

The contents of 16 elements (Al, As, Ba, Cd, Co, Cu, Cr, Pb, Fe, Mg, Mn, Mo, Ni, Se, V, and Zn) were quantified using ICP OES (Thermo Fisher Scientific, Bremen, Germany, model iCAP 6300 Duo). Instrumental and operational parameters for ICP OES are shown in Table 4.

An analyte addition and recovery test (spike test) was performed, in which 0.15 mL of analytes were added to 0.5 mL of a sample of lubricating eye drops. Spike-and-recovery and linearity-of-dilution experiments are important methods for validating and assessing the accuracy of ICP OES. Spike and recovery are used to determine whether analyte detection is affected by differences in the standard curve diluent and organic sample matrix.

### 4.5. Calibration Curves

Elementary standard stock solutions of 100 mg/L of Al, Co, Ca, Cr, Cu, Fe, K, Mg, Mn, Na, Ni, P, Se, and Zn (Specsol, São Paulo, Brazil) were utilized. In addition, calibration curves for all analytes were obtained using eleven different concentrations in the range from 0.001 ppm to 2 ppm.

The LOD and LOQ followed the analytical standards established by the IUPAC [45]. For each element detected, the LOD, LOQ, and R^2^ values were determined.

### 4.6. Determination of the Maximum Daily Dose of Lubricating Eye Drops

For the maximum daily dose (mL/day) of lubricating eye drop instilled in the eyes of an adult person, it was considered that it could be obtained through multiplying the volume of 2 drops (equivalent to 1 drop in each eye) by the maximum frequency of daily instillation (5 times a day) (See Table 5).

### 4.7. Comparative Study

Upon instillation, part of the eye drops on the ocular surface is absorbed by the cornea, conjunctiva, and sclera, and most of it goes to the systemic circulation via the mucous membrane and rhinopharynx. That is, only a small percentage of the applied dose is released into the intraocular tissues, while about 50–100% of the administered dose may reach the systemic circulation via the conjunctiva and nasolacrimal duct [98,99,100].

Parenterally administered drugs also enter the systemic circulation directly and are not subjected to the first-pass effect or the gastrointestinal environment. Therefore, concentrations of metals in the eye drops, as well as the daily application, can be compared with the values of concentrations established by the Brazilian Pharmacopoeia (BP) and ICH Q3D guideline (R2) for the parenteral route.

#### 4.7.1. Permissible Concentrations of Elemental Impurities in Lubricating Eye Drops

Since the elemental impurities (EIs) absorbed through the ophthalmic route are not considered in the pharmacopoeias and in the ICH Q3D (R2) guideline [40,41,43], in our study, the concentrations of heavy metals and metalloids quantified in the samples of lubricating eye drops were compared with the values of allowed concentrations of EIs in parenteral drugs (µg/g) established by the BP and ICH Q3D guideline (R2).

#### 4.7.2. PDE to Elemental Impurities in Ophthalmic Medications

The PDE in micrograms per day (µg/day) give the maximum permitted quantity of each element that may be, in a medication, administered through the oral, inhaled, and parenteral routes [40,41,43]. However, there are no reference values for comparing the PDE in the ophthalmic pathway.

Therefore, in our study, the daily exposure to elemental impurities (EIs) through the instillation of lubricating eye drops in the eyes (µg/day) was compared to the PDEs for parenteral drugs defined in the ICH Q3D guideline (R2). In addition, the ICH Q3D (R2) guideline allows for the use of parenterally administered PDE values without modification [40,41,42].

### 4.8. Statistical Analysis

One-way analysis of variance (ANOVA) and Tukey’s post hoc multiple comparison test were used to test for significant differences in concentrations between eye drop brands and significant differences in values of daily instillation of lubricating eye drops with and without preservatives. All statistical analyzes were performed using the GraphPad Prism 9.0 statistical package (San Diego, CA, United States). The significance level was set at *p* < 0.05.

## 5. Conclusions

In the present study, samples of 19 brands of Brazilian eye drops used to treat dry eye with and without preservatives showed different concentration values of As, Ba, Cd, Co, Cu, Mn, Mo, Ni, Se, V, Zn, Al, Cr, Fe, Mn, and Pb.

There are no concentration limit values for Al, Se, Fe, Mg, Ba, Co, and Se in drugs established by the BP and Mn defined by the ICH Q3D guideline (R^2^) for the parenteral route. However, the contents of As in the eye drops with preservatives (four different samples) and one eye drop without preservatives, as well as Cd in three eye drops with preservatives are all above the concentration value defined in the BP for parenteral use. One eye drop with preservatives had a Co concentration value greater than that established by the ICH Q3D guideline (R2) parenterally. On the other hand, the concentrations of Ba, Cr, Cu, Mn, Mo, and Ni in the eye drops were below the values established by the BP or set by the ICH Q3D (R2) parenteral guideline.

The concentrations of As, Ba, Co, Cd, Cr, Cu, Mo, Ni, V, Pb, and Se in some samples of eye drops was below the limits of the PDE set by the ICH Q3D guideline (R2).

In several countries, regulatory agencies must carry out the inspection of eye drops used in the treatment of ophthalmic diseases. Therefore, complementary studies are needed to investigate the possible risks of toxicity due to the daily instillation of lubricating eye drops containing heavy metals and metalloids.

## Figures and Tables

**Table 1:** Calibration equations (y = ax + b) *, R^2^, LOD, and LOQ obtained through external calibration.

Element	Calibration Equations y = ax + b	LOD (mg/L)	LOQ (mg/L)	R^2^
Al	y = 5875x − 44.236	0.0363	0.1211	0.9996
As	y = 449.7x + 1.2977	0.0036	0.0121	0.9996
Ba	y = 813,519x − 1440	0.0003	0.0010	0.9995
Cd	y = 14,392x − 47.211	0.0005	0.0018	0.9994
Co	y = 5674.7x − 0.31	0.0010	0.0033	0.9995
Cr	y = 17,967x + 30.812	0.0011	0.0037	0.9994
Cu	y = 22,046x + 10.232	0.0020	0.0067	0.9996
Fe	y = 10,379x + 18.352	0.0065	0.0217	0.9994
Mg	y = 381,472x + 1439	0.0017	0.0057	0.9994
Mn	y = 63,173x − 33.596	0.0002	0.0007	0.9994
Mo	y = 3475.6x − 5.3857	0.0006	0.0019	0.9993
Ni	y = 5121.1x − 4.011	0.0011	0.0038	0.9994
Pb	y = 995.99x + 9.5888	0.0050	0.0168	0.9993
Se	y = 357.79x − 3.0524	0.0045	0.0149	0.9968
V	y = 32,973x − 18.426	0.0010	0.0032	0.9995
Zn	y = 10,718x − 37.313	0.0016	0.0052	0.9989

* y = intensity; a = slope; x = concentration (mg/L); and b = intercept.

**Table 2:** Concentration of quantified metal(loid)s in lubricating eye drops compared with the permitted concentration of elemental impurities established by the BP [31] and ICH Q3D (R2) guideline for parenteral administration [40].

Element	Eye Drops 1 * (µg/g)	Eye Drops 2 * (µg/g)	Eye Drops 3 * (µg/g)	Eye Drops 4 * (µg/g)	Eye Drops 5 * (µg/g)	Eye Drops 6 * (µg/g)	Eye Drops 7 * (µg/g)	BP (µg/g)	ICH Q3D (R2) (µg/g)
Al	0.090 ± 0.020	<LOD	<LOD	<LOD	<LOD	<LOD	<LOD	NA	NA
As	0.091 ± 0.006	0.169 ± 0.009	0.077 ± 0.017	0.088 ± 0.005	0.079 ± 0.007	0.072 ± 0.008	0.108 ± 0.011	0.15	1.5
Ba	0.027 ± 0.003	0.024 ± 0.001	0.042 ± 0.001	0.021 ± 0.001	0.021 ± 0.001	0.021 ± 0.001	0.024 ± 0.002	NA	70
Cd	0.049 ± 0.001	0.051 ± 0.002	0.051 ± 0.002	0.046 ± 0.001	0.047 ± 0.002	0.048 ± 0.0005	0.048 ± 0.0005	0.05	0.2
Co	0.026 ± 0.004	0.032 ± 0.002	0.029 ± 0.004	0.014 ± 0.001	1.085 ± 0.019	0.024 ± 0.003	0.025 ± 0.005	NA	0.5
Cr	<LOD	<LOD	<LOD	<LOD	<LOD	<LOD	<LOD	2.5	110
Cu	0.015 ± 0.001	0.012 ± 0.001	0.021 ± 0.002	0.008 ± 0.001	0.004 ± 0.002	0.011 ± 0.014	0.007 ± 0.002	25	30
Fe	0.167 ± 0.009	<LOD	0.170 ± 0.020	<LOD	<LOD	0.022 ± 0.003	<LOD	NA	NA
Mg	0.315 ± 0.008	0.272 ± 0.01	0.194 ± 0.01	<LOD	8.224 ± 0.248	<LOD	5.346 ± 0.162	NA	NA
Mn	0.013 ± 0.002	0.012 ± 0.001	0.009 ± 0.001	0.007 ± 0.001	0.006 ± 0.001	0.008 ± 0.001	0.008 ± 0.001	25	NA
Mo	0.033 ± 0.002	0.043 ± 0.002	0.032 ± 0.002	0.026 ± 0.001	0.028 ± 0.003	0.029 ± 0.001	0.033 ± 0.002	2.5	150
Ni	0.040 ± 0.003	0.060 ± 0.005	0.030 ± 0.004	0.023 ± 0.002	0.026 ± 0.003	0.028 ± 0.002	0.048 ± 0.002	2.5	2
Pb	<LOD	0.032 ± 0.006	<LOD	<LOD	<LOD	<LOD	<LOD	0.1	0.5
Se	0.245 ± 0.007	0.303 ± 0.01	0.256 ± 0.013	0.248 ± 0.006	0.245 ± 0.014	0.229 ± 0.008	0.261 ± 0.01	NA	8
V	0.014 ± 0.001	0.017 ± 0.005	0.018 ± 0.003	0.014. ± 0.005	0.029 ± 0.003	0.011 ± 0.004	0.026 ± 0.007	2.5	1
Zn	0.246 ± 0.004	0.087 ± 0.003	0.066 ± 0.002	0.052 ± 0.001	0.037 ± 0.002	0.059 ± 0.003	0.065 ± 0.001	NA	NA
**Element**	Eye Drops 8 * (µg/g)	Eye Drops 9 * (µg/g)	Eye Drops 10 * (µg/g)	Eye Drops 11 * **(µg/g)**	Eye Drops 12 * (µg/g)	Eye Drops 13 * (µg/g)		**BP** **(µg/g)**	**ICH Q3D (R2) (µg/g)**
Al	<LOD	<LOD	<LOD	<LOD	0.934 ± 0.109	1.255 ± 0.034		NA	NA
As	0.204 ± 0.008	0.195 ± 0.004	0.184 ± 0.004	0.124 ± 0.002	0.147 ± 0.004	0.147 ± 0.008		0.15	1.5
Ba	0.028 ± 0.001	0.031 ± 0.002	0.025 ± 0.001	0.023 ± 0.002	0.025 ± 0.00	0.023 ± 0.001		NA	70
Cd	0.049 ± 0.001	0.051 ± 0.001	0.049 ± 0.001	0.045 ± 0.002	0.049 ± 0.002	0.046 ± 0.002		0.05	0.2
Co	0.031 ± 0.002	0.037 ± 0.002	0.029 ± 0.003	0.026 ± 0.007	0.025 ± 0.005	0.019 ± 0.002		NA	0.5
Cr	<LOD	0.004 ± 0.001	<LOD	0.004 ± 0.001	<LOD	<LOD		2.5	110
Cu	0.015 ± 0.003	0.016 ± 0.002	0.008 ± 0.001	0.003 ± 0.001	0.005 ± 0.002	0.005 ± 0.001		25	30
Fe	0.013 ± 0.004	<LOD	<LOD	<LOD	0.31 ± 0.022	0.427 ± 0.026		NA	NA
Mg	0.653 ± 0.036	24.284 ± 0.769	0.489 ± 0.024	6.004 ± 0.224	5.431 ± 0.178	0.258 ± 0.016		NA	NA
Mn	0.014 ± 0.001	0.014 ± 0.001	0.011 ± 0.0003	0.008 ± 0.001	0.01 ± 0.0004	0.012 ± 0.001		25	NA
Mo	0.043 ± 0.001	0.046 ± 0.001	0.044 ± 0.002	0.035 ± 0.002	0.037 ± 0.001	0.033 ± 0.001		2.5	150
Ni	0.071 ± 0.002	0.065 ± 0.001	0.067 ± 0.003	0.047 ± 0.002	0.056 ± 0.004	0.052 ± 0.004		2.5	2
Pb	0.019 ± 0.003	0.049 ± 0.011	0.037 ± 0.012	0.009 ± 0.001	0.013 ± 0.006	0.015 ± 0.0002		0.1	0.5
Se	0.311 ± 0.013	0.358 ± 0.006	0.317 ± 0.01	0.268 ± 0.014	0.308 ± 0.003	0.299 ± 0.018		NA	8
V	0.018 ± 0.004	0.083 ± 0.005	0.017 ± 0.002	0.022 ± 0.006	0.055 ± 0.004	0.043 ± 0.003		2.5	1
Zn	0.219 ± 0.001	0.058 ± 0.001	0.186 ± 0.002	0.552 ± 0.008	0.049 ± 0.001	0.200 ± 0.002		NA	NA
**Element**	Eye Drops 14 * (µg/g)	Eye Drops 15 ** (µg/g)	Eye Drops 16 ** (µg/g)	Eye Drops 17 ** (µg/g)	Eye Drops 18 ** (µg/g)	Eye Drops 19 ** (µg/g)		**BP** **(µg/g)**	**ICH Q3D (R2) (µg/g)**
Al	0.073 ± 0.025	0.134 ± 0.039	<LOD	<LOD	2.382 ± 0.219	<LOD		NA	NA
As	0.129 ± 0.001	0.075 ± 0.003	0.178 ± 0.002	0.108 ± 0.009	0.036 ± 0.013	0.122 ± 0.008		0.15	1.5
Ba	0.021 ± 0.001	0.021 ± 0.001	0.039 ± 0.001	0.056 ± 0.002	0.021 ± 0.002	0.03 ± 0.001		NA	70
Cd	0.044 ± 0.001	0.049 ± 0.001	0.048 ± 0.002	0.044 ± 0.002	0.047 ± 0.001	0.046 ± 0.001		0.05	0.2
Co	0.013 ± 0.001	0.028 ± 0.003	0.021 ± 0.003	0.015 ± 0.004	0.028 ± 0.009	0.031 ± 0.002		NA	0.5
Cr	<LOD	<LOD	<LOD	<LOD	<LOD	0.020 ± 0.003		2.5	110
Cu	0.002 ± 0.001	0.008 ± 0.003	0.006 ± 0.003	0.023 ± 0.006	0.011 ± 0.009	0.006 ± 0.002		25	30
Fe	<LOD	0.076 ± 0.005	<LOD	<LOD	0.453 ± 0.032	0.051 ± 0.011		NA	NA
Mg	0.225 ± 0.009	0.021 ± 0.007	<LOD	<LOD	0.124 ± 0.014	<LOD		NA	NA
Mn	0.009 ± 0.0003	0.008 ± 0.0004	0.007 ± 0.001	0.007 ± 0.001	0.011 ± 0.001	0.008 ± 0.001		25	NA
Mo	0.03 ± 0.002	0.027 ± 0.002	0.032 ± 0.002	0.026 ± 0.001	0.025 ± 0.002	0.029 ± 0.001		2.5	150
Ni	0.034 ± 0.001	0.027 ± 0.003	0.043 ± 0.004	0.026 ± 0.004	0.031 ± 0.003	0.03 ± 0.002		2.5	2
Pb	<LOD	<LOD	<LOD	<LOD	<LOD	<LOD		0.1	0.5
Se	0.278 ± 0.011	0.250 ± 0.004	0.365 ± 0.01	0.277 ± 0.011	0.211 ± 0.009	0.295 ± 0.001		NA	8
V	0.008 ± 0.002	0.014 ± 0.004	0.009 ± 0.004	0.011 ± 0.008	0.026 ± 0.007	0.011 ± 0.003		2.5	1
Zn	0.034 ± 0.002	0.019 ± 0.002	0.022 ± 0.002	0.024 ± 0.001	0.039 ± 0.002	0.136 ± 0.003		NA	NA

Notes: data are presented as mean ± standard deviation. * With preservative; ** without preservative; <LOD—analyte concentrations were below detection limits; and NA—not applicable.

**Table 3:** Values of daily instillation of lubricating eye drops compared with the PDE of ICH Q3D (R2) for parenteral administration [40].

Element	Eye Drops 1 * (µg/day)	Eye Drops 2 * (µg/day)	Eye Drops 3 * (µg/day)		Eye Drops 4 * (µg/day)	Eye Drops 5 * (µg/day)	Eye Drops 6 * (µg/day)	PDE of ICH Q3D (R2) for Parenteral Administration (µg/day)
Al	0.041	<LOD	<LOD		<LOD	<LOD	<LOD	NA
As	0.041	0.069	0.023		0.035	0.040	0.033	15
Ba	0.012	0.010	0.013		0.009	0.010	0.010	700
Cd	0.022	0.021	0.016		0.018	0.024	0.022	2
Co	0.012	0.013	0.009		0.006	0.554	0.011	5
Cr	<LOD	<LOD	<LOD		<LOD	<LOD	<LOD	1100
Cu	0.007	0.005	0.006		0.003	0.002	0.005	300
Fe	0.076	<LOD	0.052		<LOD	<LOD	0.010	NA
Mg	0.143	0.111	0.060		<LOD	4.194	<LOD	NA
Mn	0.006	0.005	0.003		0.003	0.003	0.003	NA
Mo	0.015	0.017	0.010		0.010	0.014	0.013	1500
Ni	0.018	0.024	0.009		0.009	0.013	0.012	20
Pb	<LOD	0.013	<LOD		<LOD	<LOD	<LOD	5
Se	0.111	0.124	0.078		0.099	0.125	0.104	80
V	0.007	0.007	0.005		0.006	0.015	0.005	10
Zn	0.111	0.036	0.020		0.021	0.019	0.027	NA
**Element**	Eye Drops 7 * (µg/day)	Eye Drops 8 * (µg/day)	Eye Drops 9 * (µg/day)		Eye Drops 10 * (µg/day)	Eye Drops 11 * (µg/day)	Eye Drops 12* (µg/day)	**PDE of ICH Q3D (R2) for Parenteral** **Administration (µg/day)**
Al	<LOD	<LOD	<LOD		<LOD	<LOD	0.570	NA
As	0.057	0.100	0.088		0.109	0.059	0.090	15
Ba	0.013	0.014	0.014		0.015	0.011	0.015	700
Cd	0.025	0.024	0.023		0.029	0.021	0.030	2
Co	0.013	0.015	0.017		0.017	0.013	0.015	5
Cr	<LOD	<LOD	0.002		<LOD	0.002	<LOD	1100
Cu	0.004	0.007	0.007		0.005	0.001	0.003	300
Fe	<LOD	0.006	<LOD		<LOD	<LOD	0.189	NA
Mg	2.812	0.320	11.025		0.288	2.882	3.313	NA
Mn	0.004	0.007	0.006		0.006	0.004	0.006	NA
Mo	0.017	0.021	0.021		0.026	0.017	0.023	1500
Ni	0.025	0.035	0.029		0.040	0.023	0.034	20
Pb	<LOD	0.009	0.022		0.022	0.004	0.008	5
Se	0.137	0.152	0.163		0.187	0.128	0.188	80
V	0.014	0.009	0.038		0.010	0.010	0.034	10
Zn	0.034	0.107	0.026		0.110	0.265	0.030	NA
**Element**	Eye Drops 13 * (µg/day)	Eye Drops 14 * (µg/day)	Eye Drops 15 ** (µg/day)	Eye Drops 16 ** (µg/day)	Eye Drops 17 ** (µg/day)	Eye Drops 18 ** (µg/day)	Eye Drops 19 ** (µg/day)	**PDE of ICH Q3D (R2) for Parenteral** **Administration (µg/day)**
Al	0.753	0.037	0.066	<LOD	<LOD	0.793	<LOD	NA
As	0.088	0.066	0.037	0.036	0.022	0.012	0.024	15
Ba	0.014	0.011	0.010	0.008	0.011	0.007	0.006	700
Cd	0.028	0.022	0.024	0.009	0.009	0.016	0.009	2
Co	0.011	0.007	0.014	0.004	0.003	0.009	0.006	5
Cr	<LOD	<LOD	<LOD	<LOD	<LOD	<LOD	0.004	1100
Cu	0.003	0.001	0.004	0.001	0.005	0.004	0.001	300
Fe	0.256	<LOD	0.037	<LOD	<LOD	0.151	0.010	NA
Mg	0.155	0.115	0.010	<LOD	<LOD	0.041	<LOD	NA
Mn	0.007	0.004	0.004	0.001	0.001	0.004	0.002	NA
Mo	0.020	0.016	0.013	0.006	0.005	0.008	0.006	1500
Ni	0.031	0.018	0.013	0.009	0.005	0.010	0.006	20
Pb	0.009	<LOD	<LOD	<LOD	<LOD	<LOD	<LOD	5
Se	0.179	0.142	0.122	0.073	0.055	0.070	0.059	80
V	0.026	0.004	0.007	0.002	0.002	0.009	0.002	10
Zn	0.120	0.018	0.009	0.004	0.005	0.013	0.027	NA

Notes: data are presented as mean. * With preservative; ** without preservative; <LOD—analyte concentrations were below detection limits; and NA—not applicable.

**Table 4:** ICP OES instrumental parameters.

Parameter	Setting
RF power	1250 W
Sample flow	0.45 L/min
Plasma flow rate	12 L/min
Integration time	5 s
Stabilization time	20 s
Nebulization pressure	30 psi
Plasma view	Axial
Analytes/λ (nm)	Al (309.271); As (189.042); Ba (455.403); Cd (228.802); Co (228.616); Cr (283.563); Cu (324.754); Fe (259.940); Mg (279.553); Mn (257.610); Mo (202.030); Ni (221.647); Pb (220.353); Se (196.090); V (309.311); and Zn (213.856).

**Table 5:** Lubricating eye drops with (yes) or without (no) preservatives, volume of one drop (mL), and maximum daily frequency of instillations in the eyes and maximum daily dose (mL) (as established in the package inserts).

Eye Drops	Preservative	Volume: One Drop (mL)	Maximum Daily Frequency	Maximum Daily Dose (Two Eyes) (mL/day)
1	Yes	0.045	5 *	0.454
2	Yes	0.051	4	0.408
3	Yes	0.051	3	0.306
4	Yes	0.040	5	0.400
5	Yes	0.051	5	0.510
6	Yes	0.045	5 *	0.454
7	Yes	0.053	5 *	0.526
8	Yes	0.049	5 *	0.490
9	Yes	0.045	5 *	0.454
10	Yes	0.059	5 *	0.589
11	Yes	0.048	5 *	0.480
12	Yes	0.061	5 *	0.610
13	Yes	0.060	5 *	0.600
14	Yes	0.051	5 *	0.510
15	No	0.049	5 *	0.490
16	No	0.033	3	0.200
17	No	0.033	3	0.200
18	No	0.042	4	0.333
19	No	0.033	3	0.200

* The lubricating eye drop package insert does not contain the maximum daily frequency of instillations in the eyes.

## Data Availability

The data used to support the findings of this study are available from the corresponding author upon request.

## Supplementary Materials

The following supporting information can be downloaded at: https://www.mdpi.com/article/10.3390/molecules28186508/s1, Table S1: Statistical test used in view of the results of Table 2 on the concentration of quantified metal(loid)s in lubricating eye drops; Table S2: Statistical test used in view of the results of Table 3 on values of the daily instillation of lubricating eye drops.
