# Supplementary material for: Quantification of Metal(loid)s in Lubricating Eye Drops Used in the Treatment of Dry Eye Disease
Source: Molecules. 2023 Sep 7;28(18):6508. doi: 10.3390/molecules28186508 (PMC10536462; doi:10.3390/molecules28186508)
Supplement: Supplementary file 1 [file molecules-28-06508-s001.zip › molecules-2590769-supplementary.pdf]

molecules

MDPI

Supplementary material

Quantification of Metal(loid)s in Lubricating Eye Drops Used in the Treatment of Dry Eye Disease

Marcelo de Oliveira [1,2], Elaine S. de Pádua Melo [2], Thaís Carvalho da Silva [2], Carla Maiara Lopes Cardozo [1,2], Igor Valadares Siqueira [2], Mariana Pereira Hamaji [2], Vanessa Torres Braga [1], Luiz Fernando Taranta Martin [2], Alessandro Fonseca [2] and Valter Aragão do Nascimento [1,2,*]

[1] Graduate Program in Health and Development in the Central-West Region of Brazil, Federal University of Mato Grosso do Sul, Campo Grande 79079-900, Brazil; mdeoliveira@gmail.com (M.d.O.); nutricarlalopes@gmail.com (C.M.L.C.); nessatorresb@hotmail.com (V.T.B.)

[2] Group of Spectroscopy and Bioinformatics Applied to Biodiversity and Health, School of Medicine, Postgraduation Program in Health and Development in the Midwest Region, Faculty of Medicine, Federal University of Mato Grosso do Sul, Campo Grande 79079-900, Brazil; elainespmelo@hotmail.com (E.S.d.P.M.); tata.silva1772@gmail.com (T.C.d.S.); igorvaladares@gmail.com (I.V.S.); marihamaji@gmail.com (M.P.H.); luiztaranta@gmail.com (L.F.T.M.); acfms2005@gmail.com (A.F.)

* Correspondence: aragao60@hotmail.com

Table S1. Statistical test used in view of the results of the Table 2 on concentration of quantified metal(loid)s in lubricating eye drops.

Element	Eye drops 1*	Eye drops 2*	Eye drops 3*	Eye drops 4*	Eye drops 5*	Eye drops 6*	Eye drops 7*
Al	a	< LOD	< LOD	< LOD	< LOD	< LOD	< LOD
As	a	b	ac	acd	acde	acdef	adg
Ba	a	ab	c	bd	bde	bdef	abdefg
Cd	a	ab	abc	ad	acde	acdef	abcdefg
Co	a	ab	abc	bcd	e	abcdf	abcdfg
Cr	< LOD	< LOD	< LOD	< LOD	< LOD	< LOD	< LOD
Cu	a	ab	abc	abcd	abde	abcdef	abdefg
Fe	a	< LOD	ac	< LOD	< LOD	df	< LOD
Mg	a	ab	abc	< LOD	e	< LOD	g
Mn	a	ab	bc	cd	cde	cdef	cdefg
Mo	a	b	ac	d	acde	acdef	acefg
Ni	a	b	c	cd	cde	cdef	ag
Pb	< LOD	b	< LOD	< LOD	< LOD	< LOD	< LOD
Se	a	b	ac	acd	acde	acdef	acdeg
V	a	ab	abc	acd	bce	acdf	abcdeg
Zn	a	b	c	d	e	cdf	cdg

Element	Eye drops 8*	Eye drops 9*	Eye drops 10*	Eye drops 11*	Eye drops 12*	Eye drops 13*
Al	< LOD	< LOD	< LOD	< LOD	l	m
As	h	hi	bhij	gk	bkl	bklm
Ba	abgh	ahi	abdfghj	abdefgjk	abdefgjkl	abdefgjklm
Cd	abcdefgh	abcfghi	abcdefghij	adefghk	abcdefghijkl	abdefghjklm
Co	abcdfgh	abcfghi	abcdfghij	abcdfghijk	abcdfghijkl	abcdfghijklm
Cr	< LOD	i	< LOD	ik	< LOD	< LOD
Cu	abcdefgh	abcdefghi	abcdefghij	abdefghijk	abdefghijkl	abdefghijklm
Fe	dfh	< LOD	< LOD	< LOD	l	m
Mg	abch	i	abchj	gk	gkl	abchjm

Mn	abh	abhi	abcfgj	cdefgjk	abcdfgjkl	abchijlm
Mo	bh	bhi	bhij	acgk	acgkl	acefgklm
Ni	h	bhi	bhij	agk	bgikl	bgklm
Pb	bh	bi	bhij	hk	bhkl	bhklm
Se	bh	i	bhj	acdegk	bhjl	bhjklm
V	abcdefgh	i	abcdefghj	abcdefghjk	l	elm
Zn	h	cdfgi	j	k	dl	m
**Element**	**Eye drops 14***	**Eye drops 15****	**Eye drops 16****	**Eye drops 17****	**Eye drops 18****	**Eye drops 19****
Al	an	ano	< LOD	< LOD	r	< LOD
As	gklmn	acdefo	bijp	adgknq	r	gknqs
Ba	bdefgjklmn	bdefgjkmno	cp	q	bdefgjklmnor	ahis
Cd	defgkmn	abcdefghijklmo	abcdefghijklmnp	adefgklmnpq	abcdefghijklmnopqr	adefghjklmnopqrs
Co	abcdfgjklmn	abcdfghijklmno	abcdfghijklmnop	abcdfghijklmnopq	abcdfghijklmnopqr	abcdfghijklnopqrs
Cr	< LOD	< LOD	< LOD	< LOD	< LOD	s
Cu	abdefghjklmn	abcdefghijklmno	abdefghijklmnop	abcfhiq	abcdefghijklmnopqr	abdefghijklmnoprs
Fe	< LOD	o	< LOD	< LOD	mr	dfhos
Mg	abchjmn	abchjmno	< LOD	< LOD	abchjmnor	< LOD
Mn	cdefgjkln	cdefgjklno	cdefgknop	cdefgklnopq	abchijlmnr	cdefgjklnopqs
Mo	acdefgkmn	defno	acefklmnop	defgnoq	defoqr	acdefgmnopqrs
Ni	acefn	cefno	adgknp	cdefnoq	acdefnoqr	cdefnoqrs
Pb	< LOD	< LOD	< LOD	< LOD	< LOD	< LOD
Se	bcdgklmn	acdefgkno	ip	abcdgklmnoq	fr	bhjklmnqs
V	abcdfhjkn	abcdfghjkno	abcdfhjknop	abcdfhjknopq	abcdeghjkor	abcdfhjknopqs
Zn	en	o	op	opq	enr	s

Notes: * With preservative; ** without preservative; < LOD - Analyte concentrations were below detection limits; different letters on the same line represent statistical differences between samples ($p<0,05$) by one-way ANOVA with post hoc Tukey's test; P value in the line referring to the comparisons between the concentrations of lubricating eye drops for the elements Al, As, Ba, Cd, Co, Cr, Cu, Fe, Mn, Mo, Ni, Pb, Se, V and Zn ($p < 0.0001$), Cr and Mg ($p = 0.0001$).

Table S2. Statistical test used in view of the results of the Table 3 on values of daily instillation of lubricating eye drops.

Element	Eye drops 1*	Eye drops 2*	Eye drops 3*	Eye drops 4*	Eye drops 5*	Eye drops 6*	Eye drops 7*
Al	a	< LOD	< LOD	< LOD	< LOD	< LOD	< LOD
As	a	b	c	ad	ade	acdef	g
Ba	a	b	ac	bd	abde	bdef	acg
Cd	a	ab	c	d	ae	abf	eg
Co	a	ab	abc	abcd	de	abcdf	abcdfg
Cr	< LOD	< LOD	< LOD	< LOD	< LOD	< LOD	< LOD
Cu	a	ab	abc	abcd	abcde	abcdef	abcdefg
Fe	a	< LOD	c	< LOD	< LOD	f	< LOD
Mg	a	ab	abc	< LOD	e	< LOD	g
Mn	a	b	c	cd	cde	cdef	befg
Mo	a	b	c	cd	ae	aef	bg
Ni	a	b	c	cd	cde	cdef	bg
Pb	< LOD	b	< LOD	< LOD	< LOD	< LOD	< LOD
Se	a	ab	c	ad	ae	adf	beg
V	a	ab	abc	abcd	e	abcdf	eg
Zn	a	b	c	cd	cde	f	bg

Element	Eye drops 8*	Eye drops 9*	Eye drops 10*	Eye drops 11*	Eye drops 12*	Eye drops 13*
Al	< LOD	< LOD	< LOD	< LOD	l	m
As	h	i	hj	bgk	il	ilm
Ba	acgh	acghi	hij	abcefgk	hijl	acghijm
Cd	aegh	aefghi	j	abfik	jl	jlm
Co	abcfgh	abcfghi	abcfghij	abcdfghijk	abcfghijkl	abcdfghijklm
Cr	< LOD	i	< LOD	ik	< LOD	< LOD
Cu	abcdefgh	abcdefghi	abcdefghij	abcdefgjk	abcdefghijkl	abcdefghijklm
Fe	fh	< LOD	< LOD	< LOD	l	m
Mg	abch	i	abchj	gk	l	abchjm
Mn	ah	ahi	hij	bcdefgk	ahijl	hjlm
Mo	h	hi	j	abgk	hil	bghim

Ni	h	gi	j	bgk	hl	him
Pb	bh	bi	bij	bhk	bhkl	bhklm
Se	h	hi	j	begk	jl	ilm
V	abcdfgh	i	abcdefghj	abcdefghjk	il	m
Zn	h	fi	hj	k	fil	m
**Element**	**Eye drops 14***	**Eye drops 15****	**Eye drops 16****	**Eye drops 17****	**Eye drops 18****	**Eye drops 19****
	an	ano	< LOD	< LOD	mr	< LOD
As	bgkn	adefo	adefop	cq	qr	fqs
Ba	abcefgkln	abdefkno	bdfp	abcefgknoq	dpr	prs
Cd	abefhikn	aeghino	p	pq	cr	pqs
Co	abcdfghkmn	abcd,fghijklmno	acdfkmnp	cdfmnpq	abcdfghijklmnopqr	abcdfgkmnopqrs
Cr	< LOD	< LOD	< LOD	< LOD	< LOD	s
Cu	bcdefgjklmn	abcdefgjiklmno	bcdefgjklmnop	abcdefghijklnopq	abcdefghijklmnopqr	bcdefgjklmnopqrs
Fe	< LOD	co	< LOD	< LOD	r	fhs
Mg	abchjmn	abchjmno	< LOD	< LOD	abchjmnor	< LOD
Mn	bfgkn	bcdefgkno	p	pq	bcdefgknor	cpqs
Mo	abegkn	aefo	p	pq	cdpr	pqs
Ni	an	cdefo	cdfp	cdpq	cdefopr	cdpqs
Pb	< LOD	< LOD	< LOD	< LOD	< LOD	< LOD
Se	ghkn	abeko	cp	q	cpqr	pqrs
V	abcdfhn	abcdfhjkno	abcdfnop	abcdfnoq	abcdfghjknor	abcdfnopqs
Zn	cden	o	p	pq	or	fils

Notes: * With preservative; ** without preservative; < LOD - Analyte concentrations were below detection limits; different letters on the same line represent statistical differences between samples ($p<0,05$) by one-way ANOVA with post hoc Tukey's test; P value in the line referring to comparisons between daily exposures by instillation of lubricating eye drops for the elements Al, As, Ba, Cd, Co, Fe, Mg, Mn, Mo, Ni, Se, V and Zn ($p < 0.0001$), Cr ($p = 0.0062$), Fe ($p = 0.0002$) and Pb ($p = 0.0001$).
